# Multidisciplinary management of complicated bilateral renal artery aneurysm in a woman of childbearing age

**DOI:** 10.1093/jscr/rjy147

**Published:** 2018-07-03

**Authors:** Evaldo Favi, Roberto Cacciola, Vasantha Muthu Muthuppalaniappan, Raj Thuraisingham, Mariano Ferraresso, Carmelo Puliatti

**Affiliations:** 1 Renal Transplantation, Fondazione IRCCS Ca’ Granda, Ospedale Maggiore Policlinico, Milan, Italy; 2 Renal Transplantation, Barts Health NHS Trust, Royal London Hospital, London, UK; 3 Nephrology, Barts Health NHS Trust, Royal London Hospital, London, UK; 4 Department of Clinical Sciences and Community Health, University of Milan, Milan, Italy

## Abstract

Ruptured renal artery aneurysm (RAA) during pregnancy is a rare condition associated with high mortality rates to both the mother and the foetus. We report on a 41-year-old woman at her second trimester who presented with shock to the emergency department as a result of a ruptured left RAA. While the bleeding was successfully treated with angiographic embolization, a contralateral RAA, also at risk of rupture, was discovered. Due to its position on the artery bifurcation, this lesion was considered not suitable for interventional radiology and was therefore managed by hand-assisted retroperitoneoscopic nephrectomy, *ex-vivo* repair and autotransplantation. This was done in order to preserve renal mass and give our patient a chance of having future pregnancies without risk of rupture. Three years later, her renal function is normal, there is no evidence of recurrence, and more importantly she had two successful and uncomplicated pregnancies.

## INTRODUCTION

Renal artery aneurysm (RAA) accounts for 1% of all aneurysms and 22% of visceral aneurysms [1, 2]. Rupture during pregnancy represents a life-threatening condition [2]. If the mother can be stabilized and the chances of survival of the foetus are good, caesarean section precedes any attempts to stop the bleeding. In case of foetus loss or massive maternal haemorrhage, angiographic embolization or nephrectomy remain the safest options. Endovascular techniques have revolutionized elective management of RAA. However, surgical treatment is still indicated for selected cases.

## CASE REPORT

**Figure 1:**
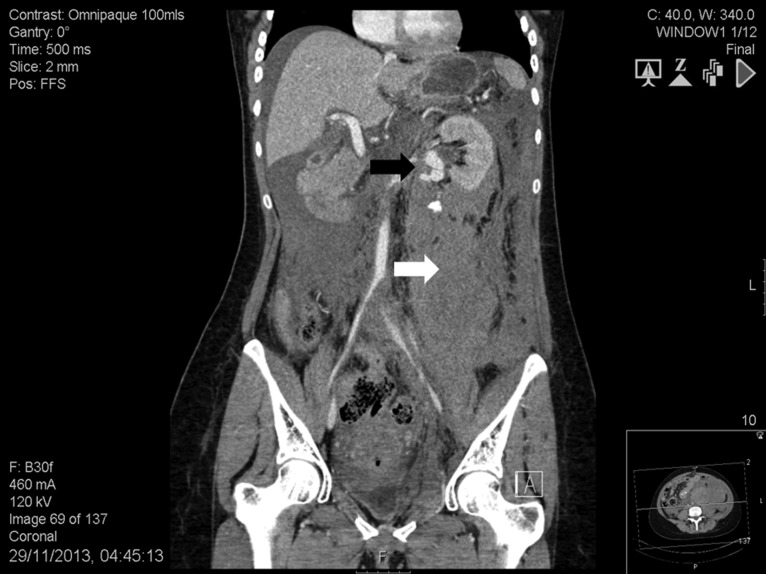
Abdomen contrast-enhanced computed tomography scan: massive retroperitoneal haematoma (white arrow) with active bleeding from a ruptured 2-cm left renal artery aneurysm (black arrow).

**Figure 2:**
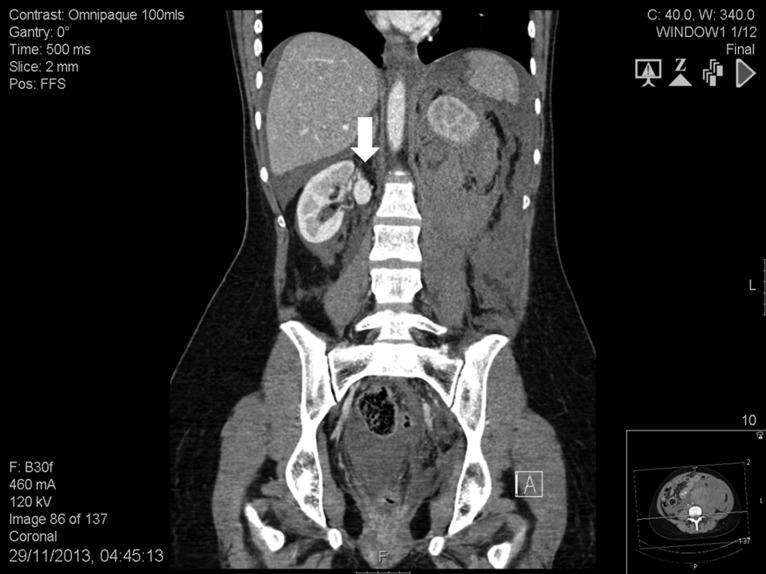
Abdomen contrast-enhanced computed tomography scan showing an intact 2.2-cm right renal artery aneurysm (white arrow).

A 41-year-old woman, 21-week pregnant, presented to the Emergency Department with left-flank pain, hypotension (100/60 mmHg) and tachycardia (95 bpm). Blood tests were: haemoglobin 7.3 g/dL, leukocytosis 23.4 cell × 10^9^/L, lactate 3.6 mmol/L, base excess −8.4 mol/L and serum creatinine 82 mmol/L. Abdominal ultrasound was normal but foetal monitoring demonstrated a drop in heart rate suggestive for impending demise. Placental abruption was suspected and following resuscitation she was brought to theatre. We found a large left-sided retroperitoneal haematoma and a non-viable foetus. Exploration also revealed a ruptured left RAA and a contralateral RAA. At this point, it was felt a percutaneous approach would have been more appropriate. Contrast-enhanced computed tomography showed a massive retroperitoneal haematoma, a ruptured left RAA (Fig. 1), and an intact right-sided RAA measuring 2.2 cm (Fig. 2). Under selective angiography, the aneurysm was embolized and the bleeding controlled [3]. Recovery was rapid but a DMSA scan performed 2 weeks later, demonstrated reduced function in the treated kidney (37%). The risk of rupture of the right RAA was deemed significant and a plan for repair was made. The lesion was saccular, wide-necked and located at the artery bifurcation thus preventing endovascular treatment. We opted for hand-assisted retroperitoneoscopic nephrectomy, *ex-vivo* repair and autotransplant.

**Figure 3:**
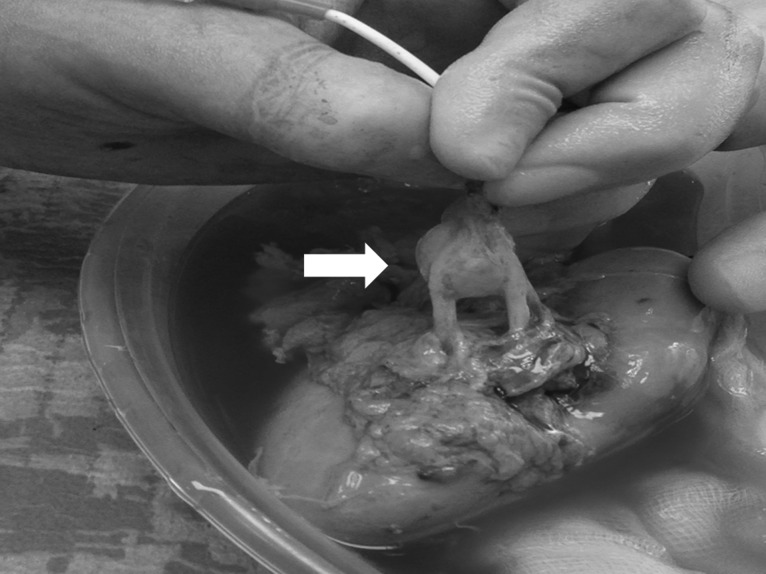
Intra-operative finding: right saccular renal artery aneurysm (white arrow) involving the main arterial branch and extending to the level of its distal bifurcation.

The patient was placed in left lateral decubitus. An 8-cm-long suprapubic incision extended to the right iliac fossa was performed and the retroperitoneum was entered. A hand-port (GelPort^®^ Laparoscopic System, Applied Medical, USA) and three 12-mm ports, one for the 30° camera and the others for the instruments were inserted. Ureter, renal artery and renal vein were divided. The kidney was extracted through the incision, flushed and immerged into a cold solution (Soltran, Baxter Healthcare, USA). The aneurysm was resected and the remaining two arteries were prepared for implantation (Fig 3). The patient was placed supine and the hand-port access was used for the autotransplant. The renal vein was anastomosed to the external iliac vein whereas the renal arteries were anastomosed to the external iliac artery. The ureteral-vesical anastomosis was performed according to Lich-Gregoire. The procedure took 481 min. Extraction, cold ischaemia and anastomosis times were 2, 48 and 52 min, respectively. Intra-operative blood loss was 280 mL. The postoperative course was uneventful. Histology showed myxoid medial degeneration of the renal artery. Three years later, her serum creatinine is 79 mmol/L with no RAA recurrence. She also completed two uncomplicated pregnancies.

## DISCUSSION

Ruptured RAA during pregnancy has been previously described [1]. Nakamura *et al.* [2] showed that over 50% of arterial aneurysm ruptures in women under 40 years are pregnancy-related. RAA is classified as saccular, fusiform, dissecting or mixed. Saccular type is the most common (70%). Aetiology includes atherosclerosis, hypertension, fibromuscular dysplasia, myxoid medial degeneration, neurofibromatosis, tuberous sclerosis, Ehlers–Danlos syndrome, infection, neoplasm, radiotherapy, surgery, trauma and chemotherapy [1]. Most patients with a RAA are asymptomatic or complain of non-specific symptoms (haematuria, hypertension, abdominal pain). Albeit rare (3%), rupture represents a catastrophic event [4]. Indications for treatment are: haemorrhage, refractory hypertension, high-flow arteriovenous fistula, embolization and high risk of rupture (enlargement, distal location, calcification, hypertension and pregnancy) [2]. Intervention is also recommended for asymptomatic lesions larger than 2 or 1 cm in women of childbearing age [5]. In case of rupture, open nephrectomy or embolization are the safest options. Successful repair has been reported but it remains anecdotal [6]. For many years, elective treatment of RAA has been mainly surgical but recent outcomes of endovascular procedures have increased the number of patients referred to interventional radiology. Major advantages are lower invasiveness and reduced morbidity [7]. However, severe complications have been described and no long-term follow-up is available. Multiple, wide-necked or distal aneurysms involving small branches of the renal artery are considered not suitable for endovascular treatment. Surgery includes nephrectomy, aorto-renal by-pass and vascular repair. Repair involves excision of the lesion and reconstruction of the artery. The procedure requires prolonged interruption of the renal blood flow with possible development of acute tubular necrosis. Cold perfusion of the kidney reduces ischaemia-reperfusion injury thus allowing time-consuming manoeuvres [8]. Back-table-surgery also gives the chance of operating without anatomical restrains. For all these reasons, nephrectomy with extracorporeal repair under cold perfusion followed by reimplantation represents the best option [9]. Open nephrectomy is considered the golden standard, but latest results support mini-invasive surgery even in complex cases. The experience gained with living donation demonstrates that laparoscopic nephrectomy is safe and offers better results than open procedures in terms of postoperative complications, pain and length of hospitalization. Renal function and long-term transplant outcomes are also equivalent [10].

Our patient was pregnant and presented with massive bleeding. The haemorrhage was managed by embolization but loss of renal function developed. Considering her strong will to have a child and the risk of rupture of the remaining aneurysm, a plan for repair was made. The lesion was unsuitable for endovascular treatment and we opted for *ex-vivo* repair and autotransplant. We preferred the retroperitoneoscopic technique because adhesions from previous surgery and anatomy altered by the organized haematoma, would have been difficult to manage using a transperitoneal approach. Moreover, the same incision for nephrectomy could be used for autotransplant without extra dissection. Hypothermic perfusion reduced possible parenchymal damage.

RAA rupture is a life-threatening condition that needs to be considered in pregnant patients with abdominal pain and hemodynamic instability. In selected cases not suitable for endovascular treatment and for functionally uninephric patients, prophylactic repair should be attempted. Hand-assisted retroperitoneoscopic nephrectomy, *ex-vivo* repair and autotransplant represents a safe and feasible option.
